# The Dependence of Global Ocean Modeling on Background Diapycnal Mixing

**DOI:** 10.1155/2014/838701

**Published:** 2014-01-22

**Authors:** Zengan Deng

**Affiliations:** ^1^Key Laboratory of Digital Ocean, State Oceanic Administration, Tianjin 300171, China; ^2^National Marine Data and Information Service, Tianjin 300171, China

## Abstract

The Argo-derived background diapycnal mixing (BDM) proposed by Deng et al. (in publish) is introduced to and applied in Hybrid Coordinate Ocean Model (HYCOM). Sensitive experiments are carried out using HYCOM to detect the responses of ocean surface temperature and Meridional Overturning Circulation (MOC) to BDM in a global context. Preliminary results show that utilizing a constant BDM, with the same order of magnitude as the realistic one, may cause significant deviation in temperature and MOC. It is found that the dependence of surface temperature and MOC on BDM is prominent. Surface temperature is decreased with the increase of BDM, because diapycnal mixing can promote the deep cold water return to the upper ocean. Comparing to the control run, more striking MOC changes can be caused by the larger variation in BDM.

## 1. Introduction

Diapycnal mixing (DM) plays a significant part in the global ocean circulation, particularly in Meridional Overturning Circulation (MOC). DM changes the water properties and contributes to MOC by lifting the deep water to the upper ocean. Therefore, determining DM accurately is vital to numerical global ocean models. Usually in global ocean models (e.g., Hybrid Coordinate Ocean Model [HYCOM], [1], and Community Climate System Model [CCSM], [2]) DM is represented by the background diapycnal mixing (BDM) in the ocean interior combined with DM algorithm (e.g., K Profile Parameterization [KPP], [3]) that resolves the added DM in the mixing intensify region (e.g., the upper ocean mixed layer).

It is believed that BDM in the ocean interior is mainly originated from the breaking of internal waves [4]. Many observation-based estimates in terms of BDM were proposed [5–9], and it is estimated that the BDM's scale of magnitude is 1 × 10^−5^ m^2^/s. However, BDM is spatial dependent; it will be heightened at high latitudes and in the regions over rough topography [10]. Currently some global models use a constant to represent BDM, such as HYCOM. However neglecting the spatial variability of BDM will result in deviations in modeling outcomes. In this work, the Argo observation-derived BDM [9] is implemented in HYCOM to improve its built-in DM parameterization, and the impacts of BDM on global ocean modeling are investigated by conducting sensitive experiments. The responses of temperature and MOC to the changes in BDM are analyzed in detail.

## 2. The Argo Observation-Derived BDM

The gridded BDM dataset for the upper 2000 m global ocean derived from Argo observations has been proposed by Deng et al. [9]. Argo project was initiated in 2000, and it had reached its goal of 3000 active floats by the end of 2007. By now more than 8000 Argo floats have been deployed by different organizations from 23 countries. Argo floats cycle to 2000 m depth every 10 days, with 4-5 year lifetimes, providing more than 100,000 temperature/salinity profiles and velocity measurements per year distributed over the global oceans. All the Argo temperature/salinity observations were collected, and then experienced the quality control procedure. Based on those refined observational data, BDM was calculated using a fine-scale parameterization adopted by Wu et al. [8].

**Figure 1:**
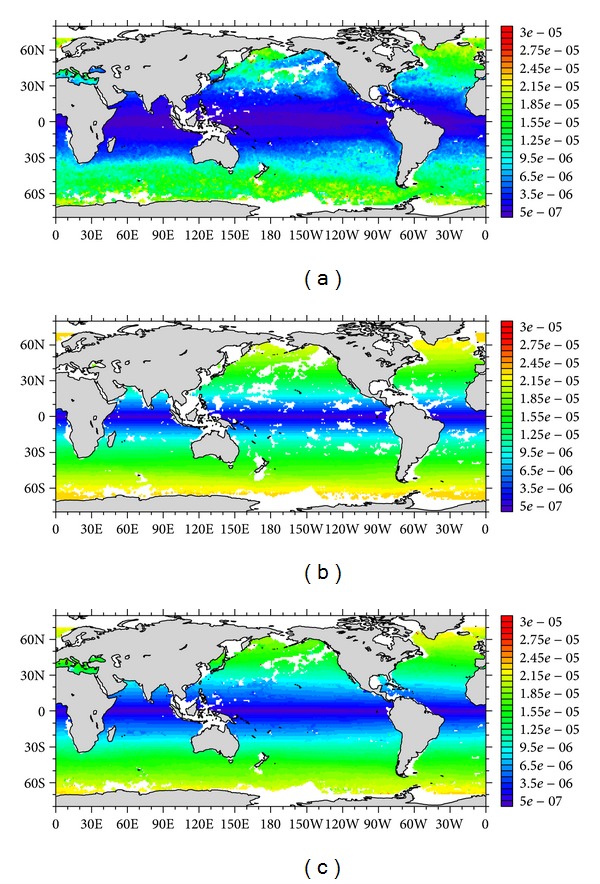
Global distribution of the BDM: (a) at depth of 112.5 m; (b) at depth of 1625 m; (c) depth averaged. (Unit: m^2^/s.) (This figure is adopted from [9].)

Figure 1 shows the global distribution of the BDM at depth of 112.5 m and 1625 m and depth-averaged BDM. DM in the upper 2000 m generally increases with the latitude, with small (large) values at the low (high) latitudes. The magnitude of DM ranges from ~0.05 × 10^−5^ to ~2.5 × 10^−5^ m^2^/s, which agrees in scale of magnitude with previous estimates. For the detailed information of the Argo-derived BDM, please refer to Deng et al. [9].

## 3. Implementation of Argo-Derived BDM in HYCOM

K Profile Parameterization (KPP) has been widely used in ocean models and is one of the DM parameterization algorithms included in HYCOM. In this study we choose the KPP scheme to parameterize the DM. Following software design description for HYCOM [11], DM (diffusivity/viscosity) consists of three components, *K* = *K* ^*s*^ + *K* ^*w*^ + *K* ^*d*^, where *K* ^*s*^ is the contribution of resolved shear instability, *K* ^*w*^ refers to BDM, and *K* ^*d*^ is the contribution of double diffusion. The parameterizations of both *K* ^*s*^ and *K* ^*d*^ are detailed in Wallcraft et al. [11]. *K* ^*w*^ is set to a constant (1 × 10^−5^ m^2^/s). As mentioned above, studies have confirmed that the variability of *K* ^*w*^ is spatial dependent; that is, it is a function of latitude and depth. So, using a constant to represent this term is obviously unsatisfactory from the physical point of view. Properly specification of *K* ^*w*^ in an ocean model can both represent physical process and ensure numerical stability [12]. For the purpose of improving the parameterization's physics, we introduce the Argo-derived BDM to replace the original constant *K* ^*w*^. Given the fact that Argo-derived BDM is only available in upper 2000 m ocean, to keep the model consistency, a depth-averaged BDM is introduced.

## 4. Experiment Settings

The configurations of global HYCOM are mainly adopted from Deng et al. [13, 14]. The calculation domain is from 64.43911°S to 64.43911°N with a resolution of 2.5° × cos⁡*φ* in latitude, where *φ* is the corresponding latitude, and from 180°W to 180°E with a resolution of 2.5° in longitude, totally 144 × 69 horizontal grid points. 26 hybrid layers are specified in the vertical direction. The initialization condition is from the Polar Science Center Hydrographic Climatology (PHC) 3.0. This Climatology is also used for lateral boundary nudging and relaxation of salinity and temperature on the ocean surface. Model was spun up for 100 years forced by ERA15 ECMWF reanalysis monthly climatology. The forcing variables are 10 m winds, ocean surface air temperature, precipitation, radiation heat flux, short-wave radiation, and water vapor mixing ratio. 24 hourly ECMWF ERA40 wind anomalies are added to ECMWF ERA15 climatology to produce the actual year run. After the spin-up run, 8 experiments with different BDM (Table 1) are designed and conducted. Exp. 5 is the control run, and Exp. 8 is the realistic run which adopts the Argo-derived BDM. The application of BDM in Exp. 8 is presented in Section 3.

## 5. Results

**Figure 2:**
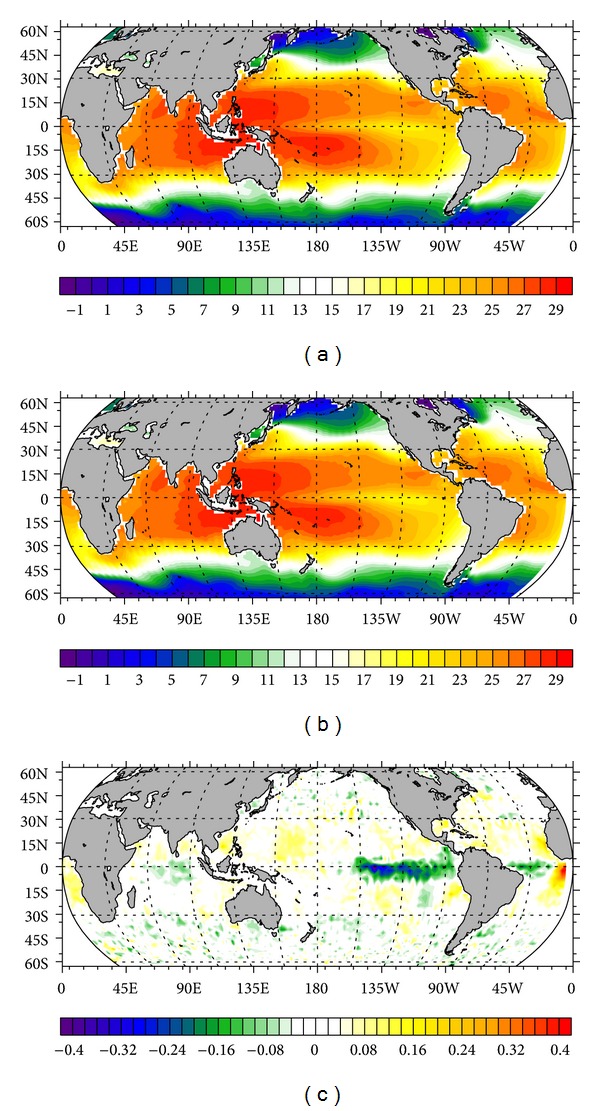
Annual-mean temperature at the ocean surface. (a) Exp. 5 (control run with default BDM of 1 × 10^−5^ m^2^/s); (b) Exp. 8 (realistic run with Argo-derived BDM); (c) differences between the Exp. 5 and Exp. 8.

**Figure 3:**
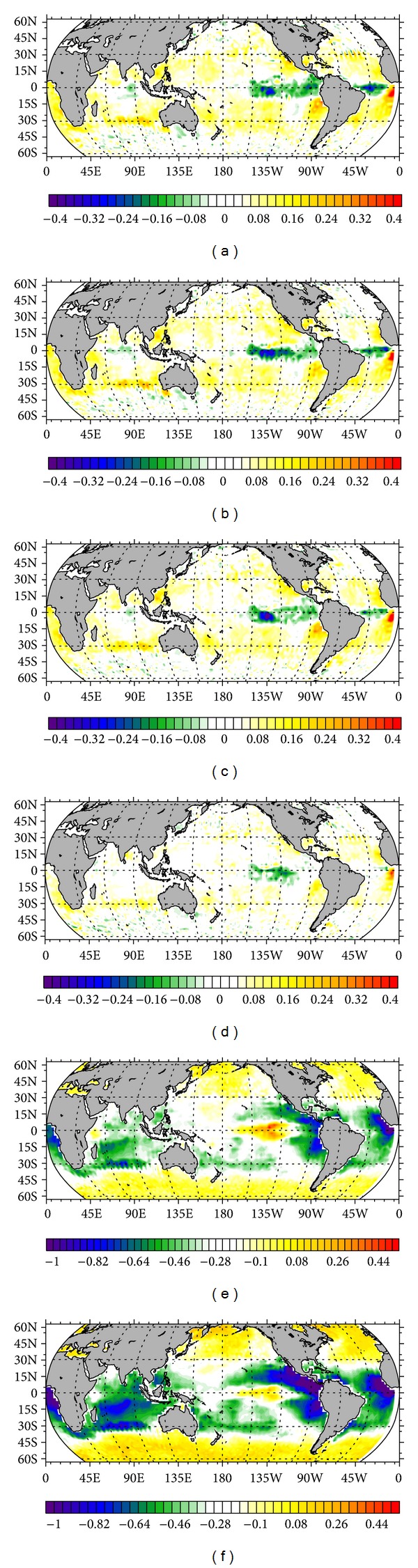
Annual-mean temperature deviation between the diagnose runs and the control run (Exp. 5). (In order from left to right and from top to bottom, the plots, respectively, present Exp. 1–Exp. 5, Exp. 2–Exp. 5, Exp. 3–Exp. 5, Exp. 4-Exp. 5, Exp. 6-Exp. 5, and Exp. 7–Exp. 5.)

The averaged temperature at the global ocean surface simulated by each experiment is listed in Table 1, showing that temperature is decreased with the increase of BDM. It can be understood that stronger mixing will lift more deep cold water to the surface and result in the decrease of surface temperature. This result agrees well with our expectation that DM is capable of promoting the transport of deep water to upper ocean in the MOC system.  Figure 2, in order from top to bottom, respectively, gives the surface temperature simulated by Exp. 5 (control run) and 8 (realistic run) and the difference between them. The distribution pattern is similar (Figures 2(a) and 2(b)), because the BDM used in those two experiments is generally in the same order of magnitude, but with no spatial variability in the control run. Some large differences appear in equatorial regions (Figure 2(c)), because in those regions the BDM of Exp. 8 (~1 × 10^−6^ m^2^/s, Figure 1) is of order smaller than that of control run (1 × 10^−5^ m^2^/s). This indicates that utilizing a constant BDM, with the same order of magnitude as the realistic one, may cause significant deviation in the simulated temperature in the equatorial area.  Figure 3 shows a similar phenomenon to that revealed by Table 1; larger BDM difference leads to more prominent temperature difference.

**Figure 4:**
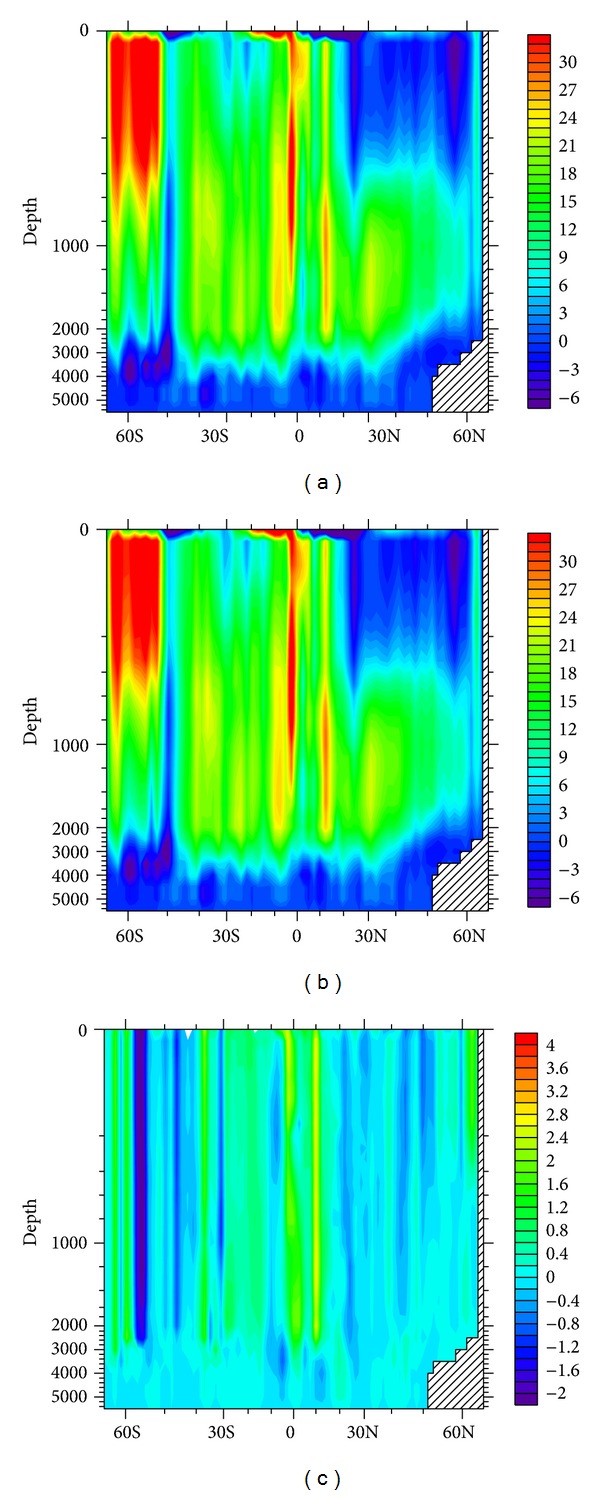
Annual-mean Atlantic MOC (units: Sv). (a) Exp. 5 (control run with default BDM of 1 × 10^−5^ m^2^/s); (b) Exp. 8 (realistic run with Argo-derived BDM); (c) differences between Exp. 5 and Exp. 8.

**Figure 5:**
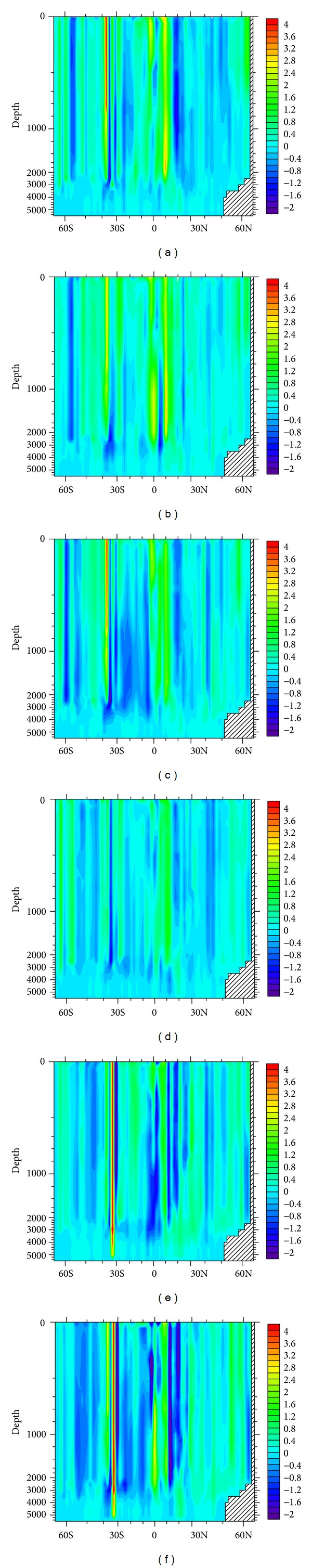
Annual-mean Atlantic MOC deviation between the diagnose runs and the control run (Exp. 5). (In order from left to right and from top to bottom, the plots, respectively, present Exp. 1–Exp. 5, Exp. 2–Exp. 5, Exp. 3–Exp. 5, Exp. 4-Exp. 5, Exp. 6-Exp. 5, and Exp. 7–Exp. 5.)

MOC in Atlantic Ocean simulated by Exp. 5 and 8 is presented by the top and middle plots in Figure 4. The similar stream function pattern is exhibited. The center of the main MOC cell locates at the depth of ~1000 m, with a maximum northward volume transport of ~30 Sv. The downwelling in the north can reach a depth of more than 3000 m. Our simulation is generally consistent with the result given by GFDL's climate model CM3 [15]. MOC is greatly altered at latitudes of 10°N and 55°S (Figure 4(c)); the changes can be as large as ~3 Sv. The alternations in the rest of regions are confined in ~1 Sv. Although BDM in Exp. 5 and 8 has the same order of magnitude, the spatial variability of BDM can cause huge difference in MOC, in particular in the tropical region and the South Ocean, because the control run's BDM is larger in tropical region and smaller in South Ocean than BDM in realistic run. Like the situation in Figure 3, Figure 5 also demonstrates that larger BDM difference results in more obvious alternation in MOC.

## 6. Conclusions

In this study, we implement the Argo-derived BDM in HYCOM and mainly focus on the sensitive experiments for detecting the responses of temperature and MOC to the changes of BDM. From the sensitive experiments, robust dependence of surface temperature and Atlantic MOC on BDM is found. Temperature is decreased with the increase of BDM, because stronger mixing will lift more deep cold water to the surface and lead to the decrease of surface temperature. It is concluded that utilizing a constant BDM, with the same order of magnitude as the realistic one, may cause significant deviation in temperature and MOC. We emphasize that updateing the constant BDM to the Argo-derived one in HYCOM will definitely improve its physics in DM parameterization and thus its modeling skill.

It is noted that BDM used in the realistic run is a depth-averaged product, thus lacking vertical variability. However, BDM has been found to be more complicated in the deep and bottom ocean; for example, it is enhanced over topographic ridges, sea mounts, and steep slopes. Therefore there is a need to introduce BDM for the whole depth with a depth-dependent one in the future. However, the absence of full-depth temperature and salinity observations over the global ocean, especially the remote open ocean, does pose a challenge for the accurate quantification of BDM in the deep ocean. At this stage, the action of replacing the fixed BDM with Argo observation-based one is a forward step.

## Figures and Tables

**Table 1:** Background diapycnal mixing and simulated temperature for the experiments.

	Exp. 1	Exp. 2	Exp. 3	Exp. 4	Exp. 5 (control)	Exp. 6	Exp. 7	Exp. 8 (realistic)
BDM (m^2^/s)	1*e* − 7	5*e* − 7	1*e* − 6	5*e* − 6	1*e* − 5	5*e* − 5	1*e* − 4	Argo-derived
Tm (°C)	15.1850	15.1848	15.1828	15.1702	15.1529	14.9952	14.7221	15.1562

BDM: background diapycnal mixing; Tm: annual-mean temperature at the ocean surface; BDM used in the control run Exp. 5 is default value, and the realistic run Exp. 8 adopts the Argo-derived BDM.
